# Clinical assessment of hypertension in children

**DOI:** 10.1186/s40885-016-0050-0

**Published:** 2016-05-17

**Authors:** Nisarg Patel, Nicole Walker

**Affiliations:** Philadelphia College of Osteopathic Medicine (PCOM), Suwanee, GA 30024 USA

**Keywords:** Pediatric Hypertension, Primary Hypertension, Secondary Hypertension, Clinical assessment, Hypertension, Children, Pediatrics, Primary practice, Cardiology, Family medicine

## Abstract

The use of blood pressure measurements have become a routine part of physical exam for the evacuation of cardiovascular health adults and, more recently, children. The most widely used definition of hypertension is delineate as greater than 90 % BP according to age, sex, and height by the National High Blood Pressure Education Program. Current research suggests that pediatric hypertension is influenced by multitude of factors including birth weight, maturity during birth, heredity, and diet leading to primary hypertension. Factors influencing secondary hypertension include renal abnormalities, coarctation of the aorta, medications, neoplasm, etc. The treatment for pediatric hypertension is carried out with diet and exercise as the first line of defense. Only under non-compliance with diet and exercise is pharmaceutical intervention appropriate. This paper outlines a concise summary of the current understanding and research for scientists, clinicians, as well as for the general population to better understand pediatric hypertension.

## Background

Blood pressure measurements for adults have proven to be crucial in the assessment of cardiovascular health, and since Stephen Hales’ experiments in the eighteenth century, have become a routine part of physical exam [1]. However, it wasn’t until 1977 that proper assessment of hypertension in children was carried out by the Task Force on Blood Pressure Control in Children [2]. Since then, numerous studies have been conducted to better understand hypertension in children. We will layout a concise summary of the current understanding and research for scientists, clinicians, as well as for the general population to better understand pediatric hypertension.

## Definition and prevalence of hypertension in children

In 2004, the National High Blood Pressure Education Program published its fourth report on hypertension in children with detailed definitions of pediatric hypertension [3]. This definition utilizes specific age, sex, and height to define the 50^th^, 90^th^, 95^th^, and 99^th^ percentile blood pressure (BP) data tables to determine normal BP. [3]. This report defines hypertension as over 90 % BP according to age, sex, and height [3].

Using the most current definition of hypertension in children, studies have found the prevalence to range between 4.7 and 19.4 % depending on a variety of factors such as age, sex, ethnicity, body mass index (BMI), parental hypertensive status, etc. [4, 5]. Hypertensive and pre-hypertensive status closely followed BMI [4]. Prevalence of hypertension was highest among Hispanics (25 %) and lowest among Asians (14 %) [5]. The explosion of obesity among children, 5 to 11 % from 1960s to 1990s, has caused a dramatic increase in hypertension among children [6]. Studies indicate that more than 20 % of children that are obese have primary hypertension [7]. This trend of increased childhood obesity will continue to have a dramatic impact on the cardiovascular health of American children and is something that clinicians must be aware off.

## Factors influencing blood pressure in children

Blood Pressure varies widely during a child’s development with an overall steadily increasing trend. The average systolic blood pressure at birth is approximately 70 mmHg which increases to 85 mmHg by one month of age [8]. Blood pressure in premature infants is considerably lower and is directly related to the weight [9]. Previous research has shown a significant inverse relationship between birth weight and risk for hypertension later in life placing premature infants in the greater risk category [10]. Starting during preschool age, children seem to be placed in a percentile distribution that they tend to follow throughout their life giving weight to the theory that essential hypertension begins in childhood [11].

As with adult hypertension, traditional separation of primary and secondary classification is useful for children. Children with secondary hypertension have a specific, potentially correctable, abnormality responsible for their high blood pressure while those with primary or essential hypertension do not.

### Primary hypertension

Primary hypertension affects millions around the world. Our inability to identify the cause of elevated blood pressure in primary hypertension undoubtedly reflects both the complex physiologic process that regulates blood pressure and the heterogeneous nature of this disorder [12].

One of the most important factors influencing primary hypertension in children is heredity [4, 9, 12, 13]. Children with paternal and/or maternal history of hypertension demonstrated significant risk for developing primary hypertension before the age of 18 [4]. There is a greater influence of heredity from paternal side than from maternal side [4]. However, other studies have found the opposite to be true leaving no significant consensus on this matter in the scientific community [9]. The inheritance pattern for hypertension seems to be multifactorial and is modified by environmental factors [13].

Diet has also been linked to primary hypertension in children. Multiple studies show a significantly higher percent of hypertensive children with non-vegetarian diet [4, 14]. Decades of research have also correlated high sodium intake and high blood pressure [4, 9, 12, 13]. The sensitivity of sodium on hypertension appears to be genetically determined as well [13].

The relationship between obesity and hypertension in children is well documented since the 80’s. Current studies indicate that overweight children and adolescents are at a substantially increased risk for several Cardiovascular Disease (CVD) risk factors. The association between obesity and high systolic blood pressure (SBP) among 2 to 9 year old children appears to be stronger than in adolescents [7]. The pathophysiology of obesity-associated hypertension has not been clearly defined. Research indicates that insulin resistance and hyperinsulinemia might contribute to enhanced renal sodium reabsorption or increasing sympathetic nervous system activity [15].

### Secondary hypertension

Secondary hypertension is accompanied with pathology or medications that could be correlated to the hypertension. Secondary hypertension is more common than primary hypertension in infants and young children. In fact, up to 85 % of children with hypertension have an identifiable cause [16]. It is the recommendation that all children with confirmed hypertension be evaluated for underlying causes to include renal parenchymal disease [16]. Table 1 cites common causes of secondary hypertension in children [12, 13, 17].

**Table 1:** Most common causes of secondary hypertension by age

Age group	Etiology
Newborn – First year	Renal artery or venous thrombosis
	Renal artery stenosis
	Congenital renal abnormalities
	Coarctation of the aorta
	Bronchoplumonary dysplasia
	Renovasular disease
	Renal parenchymal disease
	Iatrogenic
	Tumor
First to 6 years	Renal parenchymal disease
	Renovascular disease
	Coarctation of the aorta
	Tumor
	Endocrine causes
	Iatrogenic
	Essential hypertension
Age 6 to 10 years	Renal parenchymal disease
	Essential hypertension
	Renovascular disease
	Coartctation of the aorta
	Endocrine causes
	Tumor
Age 10 to 18 years	Essential hypertension
	Iatrogenic
	Renal parenchymal disease
	Endocrine causes
	Coartctation of the aorta

The causes of hypertension vary with age. Renal artery thrombosis or stenosis, congenital renal malformations, or coarctation of aorta present with newborn hypertension [13]. While these conditions present with obvious signs and symptoms, studies indicate a significant delay in the diagnosis due to improper assessment of blood pressure by physicians [12]. Children older than 6 years of age, renal artery stenosis and renal parenchymal are the leading definable causes of diastolic blood pressure measurements in excess of 90 to 100 mmHg [12]. Approximately 75–80 % of children with secondary hypertension have a renal abnormality [18]. In adolescent females, oral contraceptives have been associated with hypertension. The stimulation of renin-angiotension-aldosterone system as well as other related pathologies leading to secondary hypertension are listed in Table 1 [19].

## Ambulatory blood pressure monitoring

Traditionally, blood pressure measurements have been taken while the patient was at the office, but these practices only demonstrate the pressure in a certain setting. In children, these recorded blood pressures can be vastly different from when the child is at home or during their daily routines. Ambulatory blood pressure monitoring (ABPM) can show blood pressures at various times throughout the day and in various settings ([20].) Because ABPM can allow the clinician to get an accurate pattern of blood pressure in the patient, ABPM can be helpful in predicting target-organ damage, and may be a more accurate blood pressure measurement [20, 21]. In healthy children, ABPM has demonstrated significantly improved daytime SBP and DBP, along with a lower HR, as a result of a simple breathing meditation intervention [21]. While multiple single-center case series have demonstrated the usefulness of ABPM in identifying persistently hypertensive youths with normal clinic BP, large-scale pharmacologic trials in children utilizing ABPM have not been reported [21].

## Hypertension and target-organ damage

Prolonged hypertension can impact many organs if left unresolved. Organs and structures at risk are: heart, kidney, vascular walls, and the nervous system [22]. Childhood and adolescent hypertension is linked to future target-organ damage [21]. Target-Organ damage can include, but is not limited to carotid intima-media thickness, left ventricular mass, arterial stiffness, and left ventricle hypertrophy [21]. In children and adolescents with hypertension, it is essential to assess target-organs for any changes [22].

## Symptoms

Children with hypertension rarely have clinical evidence of the disease. The increased blood pressure is usually detected during a normal examination or before a physical examination for sports participation. Children with secondary hypertension present as asymptomatic unless the pressure is sustained or raising rapidly [18]. Clinical manifestations of underlying diseases include such things as failure to thrive and failure to grow [18]. Significant elevation of blood pressure can lead to headaches, dizziness, visual changes, nausea, epistasis, and seizures [13].

## Treatment

Once a definitive diagnosis of pre-hypertension or hypertension has been established, a treatment regimen must be outlined. As previously mentioned, increased BMI is closely related to hypertension, so if the hypertensive child is obese, a weight loss plan should be established [23]. Weight loss plans can include increased physical activity, diet modification, and behavioral therapies [23].

If diet and lifestyle changes fail to resolve hypertension after more than 6 months, this could be an indication that additional pharmacologic treatment is needed [24]. Other indications for pharmacologic treatment in adolescents include diabetes mellitus, chronic kidney disease, and stage II hypertension [24]. The use of pharmacologic treatment in pediatrics is controversial and should only be used when diet and exercise were not successful. Primary medications to consider for first line treatment are: angiotensin-converting enzyme (ACE) inhibitor, calcium channel blocker, angiotensin II receptor blocker (ARB), diuretic, and beta blocker drug classes [24].

If a secondary pharmacologic therapy is needed, the selection of this medication will need to be based on the primary medication chosen due to medication interactions and comorbidities [24].

## Conclusion

Hypertension in children and adolescents is a rising health concern that should be taken seriously and diagnosed early. With obesity on the rise, we can only conclude that the issue of prehypertension and hypertension will increase as well. With the introduction of increased physical activity and a well balanced diet, we are optimistic that obesity and thus hypertension can be treated without the need for additional pharmacologic intervention.

## Abbreviations

ACE: angiotensin converting enzyme
ARB: angiotensin II receptor blocker
BMI: body mass index
BP: blood pressure
CVD: cardiovascular disease
SBP: systolic blood pressure
